# FFLO Superfluids in 2D Spin-Orbit Coupled Fermi Gases

**DOI:** 10.1038/srep06535

**Published:** 2014-10-07

**Authors:** Zhen Zheng, Ming Gong, Yichao Zhang, Xubo Zou, Chuanwei Zhang, Guangcan Guo

**Affiliations:** 1 Key Laboratory of Quantum Information, University of Science and Technology of China, Hefei, Anhui, 230026, China; 2 Department of Physics and Centre for Quantum Coherence, The Chinese University of Hong Kong, Shatin, N.T., Hong Kong, China; 3 Department of Physics, The University of Texas at Dallas, Richardson, TX, 75080 USA and; 4 Synergetic Innovation Center of Quantum Information and Quantum Physics, USTC, Hefei, Anhui 230026, China

## Abstract

We show that the combination of spin-orbit coupling and in-plane Zeeman field in a two-dimensional degenerate Fermi gas can lead to a larger parameter region for Fulde-Ferrell-Larkin-Ovchinnikov (FFLO) phases than that using spin-imbalanced Fermi gases. The resulting FFLO superfluids are also more stable due to the enhanced energy difference between FFLO and conventional Bardeen-Cooper-Schrieffer (BCS) excited states. We clarify the crucial role of the symmetry of Fermi surface on the formation of finite momentum pairing. The phase diagram for FFLO superfluids is obtained in the BCS-BEC crossover region and possible experimental observations of FFLO phases are discussed.

In 1964, just shortly after the great success of Bardeen-Cooper-Schrieffer (BCS) theory for superconductivity^1^, Fulde and Ferrell (FF)^2^, and Larkin and Ovchinnikov (LO)^3^ independently demonstrated that a new type of superconducting state, which is characterized by Cooper pairs with nonzero total momentum and spatially modulated order parameter, may exist in certain regime of a clean superconductor under a strong magnetic field. The order parameters in real space for these two superconductors read as 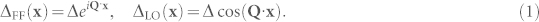
The superconducting states are now known as the FFLO superconductors or inhomogeneous superconductors. For conventional BCS cooper pairs^1^, the pairing takes place between electrons with opposite momentum and opposite spin, i.e., **k** 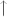 and −**k** 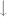. Therefore when the magnetic field exceeds certain critical value, the superconductivity is destroyed due to Pauli paramagnetic depairing effect. As a consequence, magnetism and superconductivity generally cannot coexist for the BCS type-I superconductor. The story is totally different for FFLO phases because these two different orders naturally coexist; more precisely, the FFLO phase arises from the interplay between magnetism and superconductivity. This important feature makes the FFLO phase a central concept for understanding many exotic phenomena in different physics branches, ranging from unconventional solid state superconductors^4^,^5^,^6^,^7^ (e.g., layered, heavy-fermion, organic superconductors, *etc.*), to chiral quark matter in quantum chromodynamics (QCD), and to neutron star glitches in astrophysics^8^,^9^. In the past several decades, great efforts have been made to unveil this novel quantum phase, and a lot of exotic signatures that may be related to the FFLO phase have been observed. However, until now, unambiguous and direct experimental evidences for the existence of FFLO phases are still lacking. There are several reasons for that: the existence of FFLO phase requires very stringent conditions; the direct probing of periodic oscillation of the order parameter is challenging; and the disorder effects in the superconductor induce strong scattering between different momenta that destroys the superconducting pairing^10^.

The recent experimental advances of population-imbalanced ultracold Fermi gases may have the potential to elucidate this long-sought problem. The ultracold atomic system possesses some remarkable advantages over their counterpart in solid state systems due to its high controllability and tunability^11^,^12^,^13^. The experimental parameters in ultracold atoms can be tuned in realistic experiments. Furthermore, the system can be made disorder free. On the experimental side, the superfluidity of the Fermi gas can be characterized by the generation of vortices when the gas is rotated^14^, and the momentum of the Cooper pair in the FFLO phase can be directly probed using the time-of-flight imaging^15^,^16^. Unfortunately, this system still has two major obstacles that hinder the observation of FFLO phases in recent experiments. First, the FFLO phase only exists in an extremely narrow parameter regime in 3D and 2D degenerate Fermi gases^15^,^17^, therefore in experiments the FFLO phase is generally missed out. For instance, in recent experiments with population-imbalanced Fermi gases^18^,^19^ only the phase transition from BCS superfluids to normal gases has been observed. While in another experiment^20^, the phase separation phase, which is also known as the breached pair^21^, has been observed. Second, the energy difference between the FFLO ground state and the BCS excited state is much smaller than the temperature, therefore even the parameters for the FFLO state have been reached, the Fermi gas is still too hot to reach the ground state.

The above two obstacles can be overcome using spin-orbit (SO) coupled degenerate Fermi gases with an in-plane Zeeman field. SO coupling is essential for many important phenomena in condensed matter physics^22^,^23^. In solid materials, SO coupling is induced by inversion symmetry breaking of the bulk structure^24^. While in cold atom systems, SO coupling is induced by Raman coupling between hyperfine states^25^,^26^,^27^,^28^,^29^, therefore in principle, different types of SO coupling can be created by carefully choosing different laser configurations. Experimentally, one dimensional SO coupling has been realized using Raman coupling between hyperfine states for both Bose and Fermi gases^30^,^31^,^32^,^33^,^34^,^35^, and in-plane Zeeman fields naturally exist in this system. Here we show that the combination of a Rashba-type of SO coupling and an in-plane Zeeman field can support FFLO superfluids with a unique FFLO vector in a 2D degenerate Fermi gas. The required Zeeman field or the population imbalance can be extremely small with realistic experimental parameters. The driving mechanism for the FFLO superfluid is the interplay between the deformation of Fermi surface and superconducting order^36^,^37^,^38^, thus should be in stark contrast to the physics in original FFLO superconductors^2^,^3^. In this work, we provide a comprehensive understanding for the formation of FFLO superfluids in the 2D SO coupled Fermi gas from the symmetry of the Fermi surface.

## Results

### Symmetry of Fermi Surface

The symmetry of Fermi surface is essential to understand the properties of different quantum phases and their signature in the time-of-flight imaging, which is the basic motivation of this work. The Rashba type SO coupling, *V*_so_ = *α*(*k_x_σ_y_* − *k_y_σ_x_*), is invariant under the simultaneous rotation of the momentum and spin in the *xy* plane, 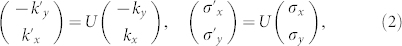
where *U* is the SO(2) rotation matrix, 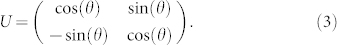
The SO(2) rotation matrix does not change the magnitude of the momentum, thus 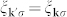 is also invariant under this transformation. Meanwhile, by defining 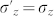, the new Pauli matrices 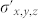 satisfy the standard commutation relation 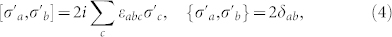
with *ε_abc_* the Levi-Civita symbol and *δ_ab_* the Kronecker delta.

The SO(2) symmetry may break down in the presence of both Rashba and Dresselhaus SO coupling. However, in this case, the Fermi surface still has the inversion symmetry, which means that the eigenvalues of single particle Hamiltonian have the basic property *E***_k_**_*σ*_ = *E*_−**k***σ*_ for any **k** and *σ*. This symmetry is unbroken by out-of-plane Zeeman field. The inversion symmetry of Fermi surface is most relevant to the physics in this work, and it is exact this symmetry ensures that the BCS phase instead of FFLO phase is more energetically favorable in the presence of out-of-plane Zeeman field. An intuitive understanding of this result is that for any state with momentum **k**, we can always find another degenerate state with opposite momentum at the same band, thus we have BCS phase. The SO coupling here plays the role of inducing pairing at the same band.

The inversion symmetry is broken by the in-plane Zeeman field because the rotation in Eq. 3 results in the following transformation, 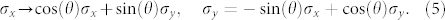
Physically, it means that it is impossible to find two degenerate states with opposite momentum at the same band. This anisotropic effect also leads to a unique FFLO vector **Q** for the FFLO superfluid, which is one of the key points of our proposal^36^. The unique **Q** makes the detection of the FFLO vector much easier in realistic experiments, see more discussions in **Measurement of the FFLO phase**. This picture is quite general and for this basic reason, the FFLO phase in this work can also be realized using other types of SO coupling^37^,^38^.

**Figure 1:**
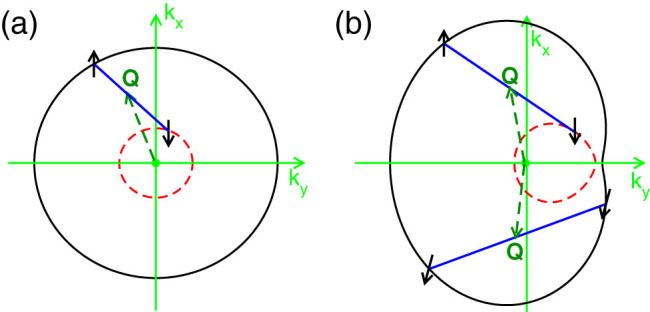
Basic physical picture for the emergence of FFLO phases in ultracold atomic systems. (a) shows the creation of FFLO phases with a Zeeman field, which is equivalent to the physics by controlling the population imbalance. The mismatched Fermi surface makes the pairing with opposite momentum and spin challenging, thus in some parameter regime the FFLO phase becomes energetically favorable. (b) shows a new mechanism for the generation of FFLO phases using the deformation of the Fermi surface. Such deformation of the Fermi surface can be constructed by an in-plane Zeeman field and SO coupling. The center of the Fermi surface is also shifted due to the Zeeman field, and as a consequence, the BCS type of pairing always becomes challenging even with a small Zeeman field. A very large FFLO phase can be observed in the parameter space. The deformation of the Fermi surface breaks the rotational symmetry of the system, thus can create the FFLO phase with a unique FFLO momentum **Q**.

**Figure 2:**
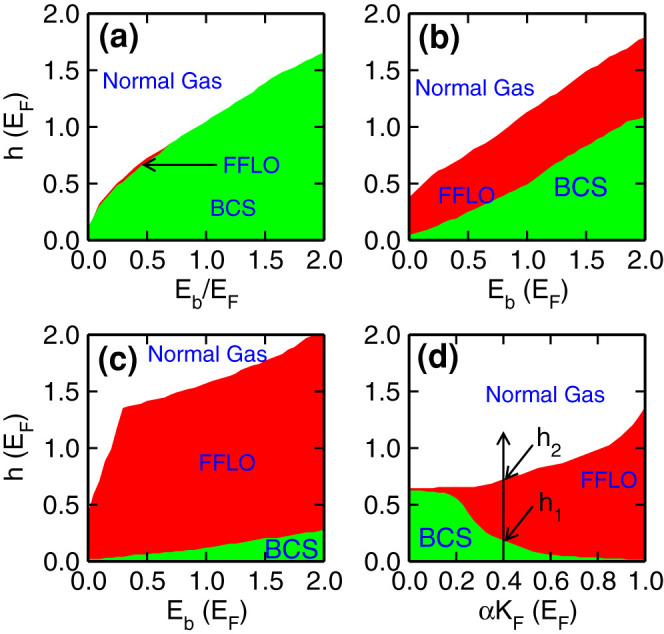
Phase diagram of the 2D degenerate Fermi gas in the presence of Rashba-type SO coupling and an in-plane Zeeman field. (a) results with vanishing SO coupling. (b), (c) correspond to the results with SO coupling *αK_F_* = 0.5 and *αK_F_* = 1.0, respectively. (d) shows the phase diagram in the *h* − *αK_F_* plane at *E_b_* = 0.4*E_F_*. *h*_1_ (*h*_2_) defines the boundary between BCS (FFLO) and FFLO (normal gas) phases.

The symmetry breaking has a direct consequence on the formation of FFLO superfluids. Before the presentation of our numerical results, we first illustrate the basic physical picture for the formation of FFLO superfluids. For the Fermi gas with only Zeeman field, see Fig. 1a, the two mismatched Fermi surfaces always form concentric circles, therefore for the s-wave pairing, the up- and down-spins acquire different Fermi momentum, *i.e.*, **k** + **Q**/2, 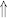 and 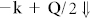, with 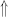 and 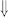 as spins in the pseudospin representation and **Q** as the total momentum of the Cooper pairs. The free energy of the system satisfies the following basic property, 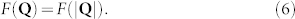
Here the Zeeman field only fixes the direction of the spin, but not the direction of the momentum axis, therefore the free energy should be invariant under the rotation of the momentum **Q**. Mathematically, it can also be understood from the fact that the total free energy depends on **k**^2^, **Q**^2^ and **k**·**Q**, thus the summation over **k** should be independent of the direction of **Q**^15^. Physically, Eq. 6 means that the total momentum of the Cooper pair can take any direction by spontaneous symmetry breaking, therefore the ground state of FFLO phases is infinity- fold degenerate. Generally in the numerical simulation, we artificially set **Q** along a particular direction and demonstrate that the FFLO phase indeed has a lower energy than the regular BCS superfluid (**Q** = 0). Due to the Pauli paramagnetic depairing effect, the FFLO phase only survives in a very narrow parameter regime, see also the numerical results in Fig. 2a. In a realistic system, any weak scattering induced by disorder effect can lead to weak coupling between the degenerate ground state manifold, making the LO superfluids, which can be regarded as a superposition of two FF superfluids with total momentum **Q** and −**Q**, as the true ground states. The LO superfluids still respect the basic symmetry argument in Eq. 6.

The physical picture is totally different when the SO coupling is presented, as schematically shown in Fig. 1b. In this case the Fermi surface is deformed and the center of the Fermi surface is no longer located at **k** = 0, therefore breaks the inversion symmetry. Here we should notice that the deformation of the Fermi surface depends strongly on the direction of the SO coupling and Zeeman field. For the model we consider here, the deformation is along the *y* direction. In the pseudospin representation (the eigenstates of single particle Hamiltonian), we have both singlet pairing and triplet pairing, where the triplet pairing will not be destroyed by a strong Zeeman field, thus the FFLO phase can be observed in a much larger parameter regime. The deformation of the Fermi surface makes the FFLO phase always energetically favorable even with a small Zeeman field. In our numerics, we find that the FFLO vector **Q** is along the deformation direction of the Fermi surface. The inversion symmetry breaking directly leads to *F*(**Q**) ≠ *F*(−**Q**), which stabilizes the FF superfluids against the formation of LO superfluid phases.

Generally, the mismatch of the Fermi surface is the basic route to the FFLO phase, and such mismatched Fermi surface can be created by population imbalance^18^,^19^,^20^, Zeeman field^17^ or mass imbalance^39^,^40^. In this work, together with our previous work^36^, we demonstrate that the FFLO phase can be created more efficiently through the deformation of the Fermi surface, which can be constructed by SO coupling, or, non-Abelian gauge field^25^,^26^,^27^,^28^,^29^, and Zeeman field. Notice that the generation of non-Abelian gauge fields is a subject of intensive investigations in ultracold atoms in the past decade, see a recent review^29^. For this new route, the Zeeman field is still needed. Otherwise the system has the time-reversal symmetry and the band structure should satisfy 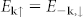, which means that two Fermions with opposite momentum on the Fermi surface can always form BCS Cooper pairs efficiently (the pairing is not necessary in the singlet channel), leading to BCS superfluids, instead of FFLO phases. Our route here, however, shows that the FFLO phase may be observed even with a small Zeeman field (thus small population imbalance). It therefore represents a new driving mechanism for FFLO superfluids.

### Phase diagram

We first present the phase diagram with different SO coupling strength and Zeeman field in Fig. 2. Without SO coupling, see Fig. 2a, we see that the FFLO phase only exists in an extremely narrow parameter regime. When *E_b_* ≥ 0.7*E_F_*, the FFLO phase disappears, thus such a phase can be only observed in the weak binding energy regime, for instance, *E_b_* ∈ (0.15, 0.7)*E_F_*. Similarly, the FFLO phase can also be observed in the 3D system^36^; however, the FFLO phase in 3D Fermi gases can only be observed near the unitary regime within a small parameter region, and the small FFLO regime can be easily missed out in realistic experiments, which is also one of the main reasons why the FFLO phases cannot be observed in recent experiments in 3D Fermi gases^18^,^19^,^20^. With an increasing SO coupling strength, see Fig. 2b for *αK_F_* = 0.5*E_F_* and Fig. 2c for *αK_F_* = 1.0*E_F_*, we find that the FFLO phase regime is greatly enlarged. In the strong SO regime in Fig. 2c, we even observe that the phase diagram is almost fully filled by the FFLO phase, while the BCS superfluid phase is greatly suppressed and only survives in a very small regime. To see the impact of SO coupling more clearly, we plot in Fig. 2d the phase diagram in the *h* − *αK_F_* plane with *E_b_* = 0.4*E_F_*. We define the boundary between BCS superfluid and FFLO phase as *h*_1_ and the boundary between FFLO phase and normal gas as *h*_2_ for convenience, see Fig. 2d. We observe *h*_1_ decreases while *h*_2_ increases with the increasing SO coupling strength, therefore the FFLO phase is greatly enlarged in the strong SO coupling regime. In the strong SO coupling region, *h*_1_ becomes very small, but never becomes zero because the the Zeeman field is essential for the FFLO phase, which breaks the time-reversal symmetry.

**Figure 3:**
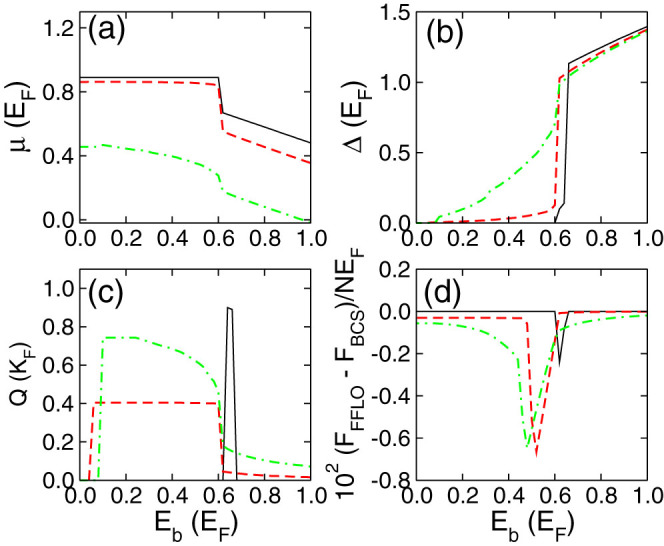
Sysmtem parameters as a function of binding energy. (a)–(c) show the evolution of chemical potential, order parameter and FFLO vector **Q** as a function of the binding energy. In (d) we plot (*F*_FFLO_ − *F*_BCS_)/*nE_F_ vs. E_b_*. In all calculations we set *h* = 0.8*E_F_*. The solid line, dashed line and dash-dotted line correspond to *αK_F_* = 0.0, 0.5 and 1.0, respectively.

We plot the evolution of chemical potential, order parameter and *Q* as a function of the binding energy in Fig. 3, where the Zeeman field is fixed to *h* = 0.8*E_F_*. As we decreases the binding energy, we observe a sudden drop of the order parameter in Fig. 3b at zero SO coupling strength due to the Pauli paramagnetic depairing effect, following which there is a small regime that supports FFLO phase, see also the solid line in Fig. 3c, *Q* ≠ 0. With the increasing SO coupling strength, we see that the change of Δ becomes a smooth function of *E_b_*, and in a much larger parameter regime we can observe the FFLO phase with a non-zero *Q*. The results in Fig. 3c clearly demonstrate the enlargement of FFLO superfluid phases observed in Fig. 2.

The FFLO superfluids in our model may be directly observed at finite temperature. We denote *F*_FFLO_ as the free energy obtained by letting **Q** as a free parameter, while *F*_BCS_ as the free energy by enforcing **Q** = 0. In the FFLO phase regime, *F*_BCS_ represents the free energy of BCS excited states, therefore the energy difference per particle between *F*_FFLO_ and *F*_BCS_, i.e., 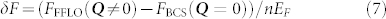
which directly characterizes the stability of the FFLO phase (i.e., the larger |*δF*|, the more stable FFLO phase). Obviously, when **Q** = 0, *δF* = 0. The numerical results are presented in Fig. 3d, where we clearly see the enhancement of *δF* due to the SO coupling. However, in 2D Fermi gases the enhanced factor is about two order of magnitude smaller than that in SO coupled 3D Fermi gases.

**Figure 4:**
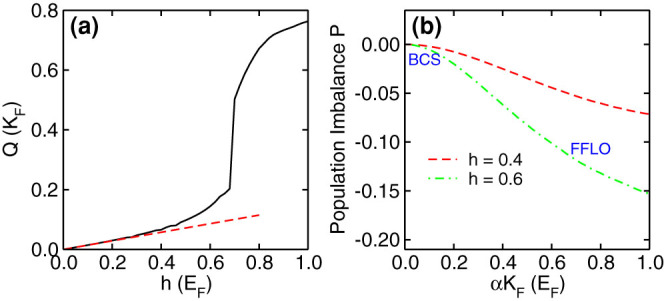
FFLO momentum and population imbalance. (a) FFLO momentum *Q* as a function of Zeeman field (solid line), and the dashed line is the linear fitting at the small Zeeman field regime, which gives Q = 0.1434h. (b) Population imbalance *P* = 〈*σ_x_*〉/*n* as a function of SO coupling strength. The parameters are: (a), *E_b_* = 0.4*E_F_*, *αK_F_* = 1.0*E_F_*; (b) *E_b_* = 0.4*E_F_*.

In Fig. 3b, we see that in the BCS superfluid regime (*E_b_* > 0.7*E_F_*), the order parameter decreases with the increasing SO coupling, which is in sharp contrast to that for SO coupled BEC-BCS crossover with Z direction Zeeman field. Generally, with the *Z* direction Zeeman field, the SO coupling plays the role of increasing the density of states near the Fermi surface, which increases the order parameter as well as the critical temperature. With an in-plane Zeeman field, the SO coupling plays a totally different role. Firstly, the in-plane Zeeman field deforms the Fermi surface, thus any small deformation leads to a small finite momentum *Q*, as shown in Fig. 4a. In the small Zeeman field regime, the momentum *Q* ∝ *h*, while in the large Zeeman field regime, it becomes a nonlinear behavior. Secondly, the SO coupling can enhance the population imbalance, see Fig. 4b, thus renders the decrease of the order parameter as observed in Fig. 3b. In the FFLO phase regime, the order parameter increases with the increasing SO coupling strength due to the formation of the FFLO phase. Notice that in our model, the FFLO superfluid can appear with extremely small population imbalance, thus it is driven by the interplay between the deformation of Fermi surface and the superconducting order, instead of the original idea of FFLO superconductors which arises from the interplay between magnetism and superconducting order. The new driving mechanism represents a more efficient way to create FFLO superfluids.

### Measurement of the FFLO phase

**Figure 5:**
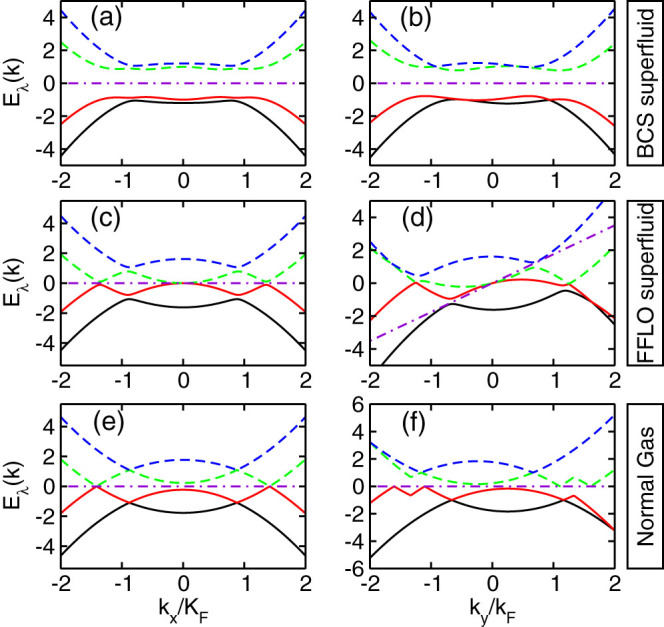
Eigenvalues *E_λ_* (*λ* = 1, 2, 3, 4) of the SO coupled degenerate Fermi gas. (a), (b) correspond to the typical eigenvalues *E_λ_* for the BCS superfluid with parameters *h* = 0.2, *E_b_* = 0.4, *αk_F_* = 1.0. (c), (d) correspond to the typical eigenvalues *E_λ_* for the FFLO superfluid with parameters *h* = 0.4, *E_b_* = 0.4, *αk_F_* = 1.0. (e), (f) correspond to the typical eigenvalues *E_λ_* for the normal gas with parameters *h* = 1.0, *E_b_* = 0.4, *αk_F_* = 1.0. The first column shows the results along the *x* direction, while the second column shows the results along the *y* direction. The energies are in unit of *E_F_*. In each panel, the dash-dotted line represent 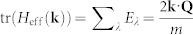.

**Figure 6:**
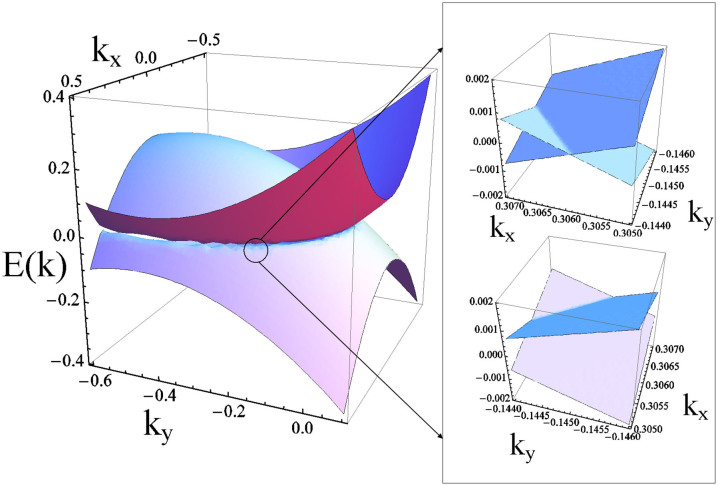
Gapless excitations for the FFLO phase. Near *E***_k_**_,*λ*_ = 0, the energy shows a clear linear dispersion. *k_x_* and *k_y_* are in unit of *K_F_*. In 2D system the linear dispersion is essential to make the FFLO phase robust against low-energy fluctuations.

The three different phases have different properties which can be used for the identification of these phases. In Fig. 5, we plot the typical band structures *E_λ_*, *λ* = 1, 2, 3, 4, for the BCS superfluid, the FFLO phase and the normal gas. Due to the rotational symmetry breaking, we have to plot the dispersions along the *k_x_* and *k_y_* axes, respectively. For a typical BCS superfluid (**Q** = 0) in Fig. 5a and Fig. 5b, we see that the system is always gapped and the band structure is always symmetric about **k** = 0 for the dispersion along *k_x_*. While along the *k_y_* axis, such symmetry is absent. In fact we can verify exactly that the BCS superfluid is always gapped. However for the FFLO phase, the superfluid becomes gapless along both *k_x_* and *k_y_* axes. Along the *k_x_* axis the band structure is symmetric about **k** = 0, but along the *k_y_* direction such symmetry is broken. For the FFLO phase we observe 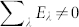 because **Q** ≠ 0 (see numerical results in Fig. 5). Note that the gapless excitation is a typical feature of the FFLO phase, as pointed out in the literature^15^. In the vicinity of the gapless excitation, see Fig. 6, the dispersion becomes linear which is essential to ensure that the FFLO phase is robust against the low-energy fluctuations. Here we should emphasize that not all FFLO phases are gapless. The FFLO state may become gapless only when *Q* is relatively large, while for a small *Q* (near the boundary between FFLO and BCS superfluid) the FFLO phase is still gapped, similar to that in the BCS superfluid. For the normal gas the band structure also shows strong deformation along the *k_y_* axis, as seen in Fig. 5e and Fig. 5f.

**Figure 7:**
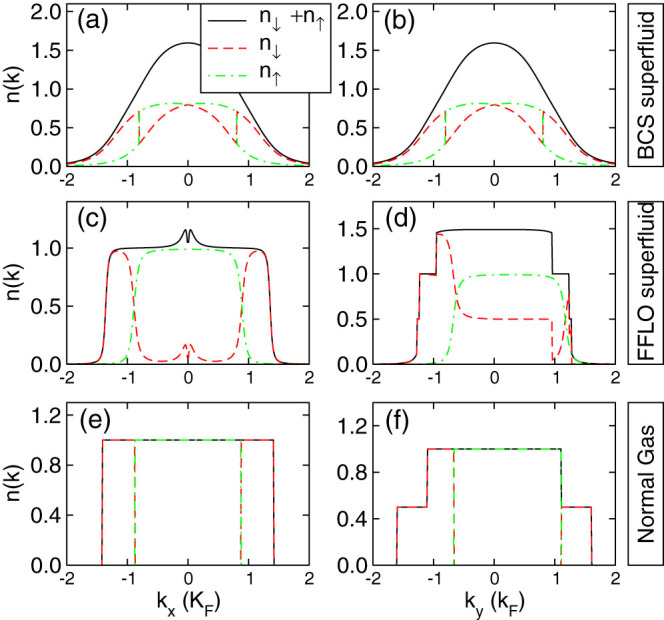
Momentum distributions. 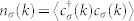
 and 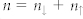 for different quantum phases. Other parameters are exactly the same as that in Fig. 5.

The corresponding momentum distributions 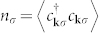 and 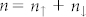 provide an important tool to detect the properties of the FFLO state because they can be directly measured via free expansion of the atomic cloud. We plot the momentum distributions in Fig. 7 for three different phases presented in Fig. 5 at zero temperature. The dispersion properties of the band structures can be directly reflected on the corresponding momentum distributions. We see that for three different quantum phases, the momentum distributions are always symmetric about **k** = 0 along the *k_x_* direction, while show strong asymmetric along the *k_y_* direction. However, the sum of the momentum distributions *n* for spin up and spin down components still shows perfect symmetry about **k** = 0 along both *k_x_* and *k_y_* directions. Therefore detecting the asymmetry of the superfluid is not sufficient for the identification of the FFLO phase. To identify the superfluid nature of the FFLO phase, we have to rotate the sample to create vortices, which is a direct evidence of superfluidity. Near the boundary between difference phases, the fluctuation effect may become significant thus the phase boundary region is not suitable for the observation of vortices. With the large FFLO phase region in our model we can safely choose some parameters in the middle of the FFLO phase region where the fluctuation effect should be minimized. The large FFLO superfluid phase ensures that it will not be missed out in future realistic experiments.

**Figure 8:**
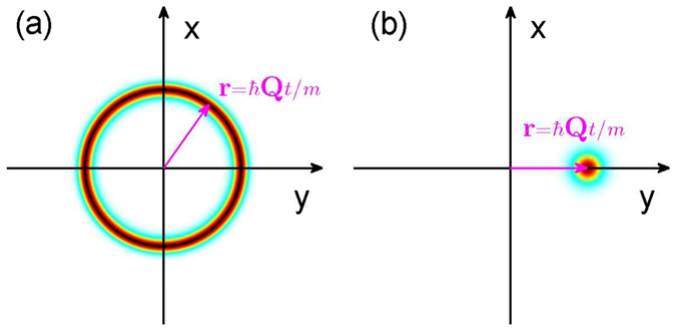
Typical time-of-flight image for the degenerate Fermi gas with (a) and without (b) inversion symmetry. For the system without inversion symmetry, the FFLO momentum **Q** is along the principle Fermi surface deformation direction, and can be directly measured in experiments.

The properties of the FFLO phase may be measured using a number of methods developed in ultracold atom systems, for instance, shot-noise correlation^43^ and density-density correlation measurement^44^,^45^, which shows a peak at the Cooper pair momentum **Q**. After released from a trapping potential, the free expansion of the Fermi cloud has a peak at **r** = ħ**Q***t*/*m*, therefore the direct measurement of the FFLO momentum **Q** is possible^46^. In our model when **Q** is unique, repeated measurement to determine the FFLO momentum becomes possible, see Fig. 8. In the FFLO phase without SO coupling, the ground state is independent of the direction of **Q**, thus only a circle with radius ħ|**Q**|*t*/*m* can be observed, see Fig. 8. So the time-of-flight imaging provides the most convenient way to probe the symmetry effect of the degenerate Fermi gas. In other words, the time-of-flight imaging directly reflects the deformation direction of the Fermi surface. The FFLO phase can also be measured using the Fourier sampling of time-of-flight images proposed by Duan^47^. The gapless excitations in the FFLO phase may be observed using the Bragg spectroscopy^46^.

## Discussion

To summarize, in this paper we study the possible FFLO phase in SO coupled degenerate Fermi gases with in-plane Zeeman fields. We show that the parameter region for the FFLO phase can be greatly enlarged due to the deformation of the Fermi surface. The emergence of the FFLO phase is explained from different angles. The properties of the BCS superfluid, FFLO phase and normal gas have also been discussed and their measurement through the time-of-flight imaging is presented. Our results indicate that the deformation of the Fermi surface provides a more efficient method to generate the FFLO phase. Because the SO coupling has been realized in Bose^30^,^31^,^32^,^33^ and Fermi^34^,^35^ cold atom gases in experiments, where the in-plane Zeeman field can be naturally created^30^,^31^,^32^,^34^,^35^ and tuned, we expect the idea in this work may provide a path for elucidating the long-standing problem about FFLO phases in experiments in the near future.

## Methods

We consider a 2D degenerate Fermi gas with Rashba-type SO coupling and an in-plane Zeeman field. The 2D degenerate Fermi gases can be constructed by applying a strong standing wave along the third direction, and have been realized in recent experiments^41^. The 2D SO coupled Fermi gases can be described as^48^,^49^

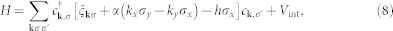
where *α* is the SO coupling strength, *σ_x_* and *σ_y_* are the Pauli operators, 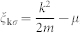, and **k** = (*k_x_*, *k_y_*). The *s*-wave scattering interaction is given by 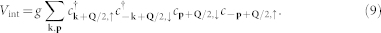
The effective scattering interaction *g* in Eq. 14 in a 2D Fermi gas should be regularized through^42^

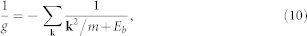
where the binding energy *E_b_* can be tuned by varying the *s*-wave scattering length through Feshbach resonance^11^,^12^,^13^.

Introducing the order parameter in the momentum space 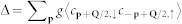, the Hamiltonian can be written as 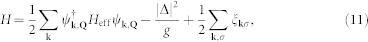
where the effective Hamiltonian reads as, 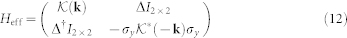
with 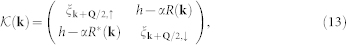
*R*(**k**) = (**k** + **Q**/2)*_x_* + *i*(**k** + **Q**/2)*_y_*, and *I*_2×2_ = diag(1, 1). The basis defined in Eq. 11 is 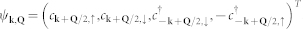.

The thermodynamical potential at zero temperature reads as 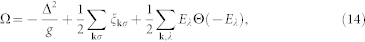
where the Heaviside step function 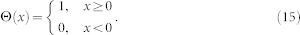
*E_λ_*, *λ* = 1, 2, 3 and 4, are the eigenvalues of the effective Hamiltonian *H*_eff_.

We use the mean field theory as the main theoretical tool in this work. The order parameter Δ, chemical potential *µ*, and the FFLO momentum **Q** should be solved self-consistently due to the conservation of atom number, i.e., 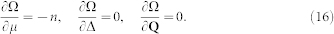
In our calculation, we choose the energy unit as the Fermi energy *E_F_* of the system without interaction, Zeeman field and SO coupling. The corresponding length scale 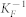 is defined through the Fermi momentum *K_F_*. At finite temperature, the 2D system does not have the long-range order due to the phase fluctuation and the relevant physics is the Kosterlitz-Thouless transition^50^. In this paper, we restrict to the physics at zero temperature, where the mean-field theory is still valid. For this specific model, we find **Q** = (0, *Q*), which means that the FFLO momentum is along the Fermi surface direction, see Fig. 1. We notice that the direction of the FFLO vector **Q** is also consistent with the results in solid state systems with weak SO coupling^51^.

## Author Contributions

Z.Z., M.G., X.Z. and C.Z. conceived the idea. Z.Z. and Y.Z. performed the calculation. M.G., X.Z. and C.Z. wrote the manuscript. M.G., X.Z., C.Z. and G.G. supervised the whole research project.

## Additional Information

**Note added**: Our manuscript was initially posted at arXiv (arXiv:1212.6826).
